# Determination of Sensitivity and Specificity of Diagnostic Nasal Endoscopy Compared to Computed Tomography Scan of Nasal and Paranasal Sinuses Among Patients Suffering From Chronic Rhinosinusitis

**DOI:** 10.7759/cureus.87760

**Published:** 2025-07-12

**Authors:** Anoop Kumar Singh, Surendra Kumar Kanaujia, Mohammad Saleem Khan

**Affiliations:** 1 Otolaryngology - Head and Neck Surgery, Ganesh Shankar Vidyarthi Memorial Medical College, Kanpur, IND

**Keywords:** chronic rhino sinusitis, ct (computed tomography) imaging, diagnostic nasal endoscopy, sensitivity, specificity

## Abstract

Background

Chronic rhinosinusitis (CRS) significantly impacts patients' quality of life. Accurate diagnosis is crucial for effective management, with diagnostic nasal endoscopy (DNE) and computed tomography (CT) being primary tools. It significantly impacts patients’ quality of life, causing symptoms like nasal congestion, facial pain, decreased sense of smell, and nasal discharge.

This study aimed to evaluate the sensitivity and specificity of DNE in comparison to CT, the gold standard, for CRS diagnosis.

Methodology

A prospective observational study was conducted at Ganesh Shankar Vidyarthi Memorial Medical College, Kanpur. A total of 109 patients presenting with CRS symptoms for more than 12 weeks were included. All participants underwent both DNE and CT of the paranasal sinuses. Correlations between radiological and endoscopic findings were evaluated to identify patterns and inform treatment decisions.

Results

A statistically significant correlation was found between endoscopic and radiological findings (χ² = 55.20, *p* < 0.00000001), indicating strong diagnostic concordance. DNE achieved a sensitivity of 93.42%, specificity of 75.75%, positive predictive value (PPV) of 89.33%, and negative predictive value (NPV) of 83.33%. These findings highlight the diagnostic utility of DNE for detecting mucosal abnormalities, while also emphasizing the superior anatomical detail provided by CT imaging.

Conclusion

DNE is a valuable, minimally invasive tool for diagnosing CRS, offering high sensitivity in detecting mucosal inflammation. However, CT remains essential for a complete anatomical assessment, particularly in complex or refractory cases.

## Introduction

Chronic rhinosinusitis (CRS) is a prevalent and often debilitating condition characterized by persistent inflammation of the nasal and paranasal sinus mucosa lasting over 12 weeks. It may or may not be associated with nasal polyposis and should be recognized regardless of treatment status. The global prevalence of CRS is estimated to be between 5% and 15% of the general population, making it one of the most prevalent chronic diseases worldwide. It significantly impacts patients' quality of life, causing symptoms like nasal congestion, facial pain, decreased sense of smell, and nasal discharge [1]. Due to its chronic nature and association with other respiratory conditions such as asthma and allergic rhinitis, CRS represents a major healthcare burden, both in terms of direct medical costs and lost productivity [2]. Given the complex anatomy of the paranasal sinuses, accurate diagnosis remains a challenge, often requiring a combination of clinical evaluation, imaging, and endoscopic examination. The 1997 Rhinosinusitis Task Force (RSTF) working definition of chronic rhinosinusitis (CRS) described it as any form of rhinosinusitis lasting for more than 12 weeks, with the presence of two or more major criteria or one major criterion and two minor criteria from a list of 12 clinical factors/symptoms [3]. These criteria included five major criteria such as facial pain or pressure, facial congestion or fullness, nasal obstruction or blockage, decreased sense of smell, discolored or purulent nasal or postnasal secretion and seven minor criteria as headache, fever, halitosis, fatigue, dental pain, cough and ear pain, pressure, or fullness.

However, this combination of major and minor clinical factors/symptoms proved cumbersome in clinical practice. To simplify the diagnosis and make it more clinically feasible, the American Academy of Otolaryngology-Head and Neck Surgery (AAO-HNS) convened another panel in 2007 [4]. CRS is generally classified into two main phenotypes: CRS with nasal polyps (CRSwNP) and CRS without nasal polyps (CRSsNP). These subtypes differ not only in their clinical presentation but also in their immunologic and inflammatory profiles. CRS is a multifactorial disease that arises from a combination of host, environmental, and microbial factors. Bacterial infections can contribute to the persistence of inflammation in the sinuses, with common pathogens including *Streptococcus pneumoniae*, *Haemophilus influenzae*, and *Moraxella catarrhalis* [5,6]. CRSwNP is typically characterized by a more severe inflammatory response, often with a Th2-dominant immune response. while CRSsNP is generally associated with a Th1-dominant response. Patients with CRSwNP, a Th2-dominant inflammatory response is commonly observed, leading to the release of pro-inflammatory cytokines such as interleukin-4 (IL-4), IL-5, and IL-13, which contribute to eosinophilic inflammation and polyp formation [7-9]. Understanding the underlying immune mechanisms in these distinct phenotypes is crucial for developing targeted therapies that address the specific inflammatory pathways involved. Anatomical variations can be congenital or acquired, and patients with such abnormalities are at increased risk for developing CRS, particularly when combined with other predisposing factors like allergies or infections. A defective CFTR gene leads to abnormal mucus secretion and impaired immune mucociliary clearance, making them highly susceptible to developing CRS [10,11].

Both diagnostic nasal endoscopy (DNE) and computed tomography (CT) are critical for assessing CRS. DNE offers direct visualization of the nasal passages, providing insights into mucosal inflammation, polyps, and purulent secretions, while CT provides detailed anatomical images of the sinuses, revealing bone changes, mucosal thickening, and opacification of sinuses on CT Imaging. Despite their complementary strengths, the relative accuracy of each method remains a topic of clinical debate, particularly in terms of their roles in surgical planning and long-term management.

## Materials and methods

This single-center, observational, and analytical study was conducted in the Department of Ear, Nose, and Throat (ENT) at Ganesh Shankar Vidyarthi Memorial (GSVM) Medical College and its affiliated Lala Lajpat Rai (LLR) Hospital, Kanpur, Uttar Pradesh, India. The study was carried out over a period of 12 months, from May 2024 to April 2025. Patients attending the ENT outpatient department (OPD) and those admitted to the ENT inpatient services at LLR Hospital were included in the study population.

This study included patients having sinusitis for more than three months in the age group 10 to 60 years, and patients with acute rhinosinusitis and a history of sinonasal trauma, sinonasal surgery, sinonasal tumor, or inverted papilloma were excluded. The study utilized a simple random sampling technique for those who met the inclusion criteria and were willing to participate, after receiving approval from the institutional ethics committee. Before commencing the study, written informed consent was obtained from the patients.

The study obtained clearance from the Ethics Committee of GSVM Medical College, Kanpur (Approval No. EC/BMHR/2024/04, dated January 10, 2024). Written informed consent was obtained from all participating patients or their legal guardians. This step was crucial to ensure that participants were fully informed about the nature of the study, the diagnostic procedures involved (including radiological and endoscopic examinations), the potential risks and benefits, and the confidentiality of the collected data.

A comprehensive medical history was taken for each patient, focusing on their diagnosis of CRS, including the duration and severity of symptoms, previous treatments, and any history of sinus or nasal surgeries. This was followed by a detailed examination of the nasal cavity, sinuses, and throat to identify any visible abnormalities, such as mucosal edema, polyps, or signs of infection. The systemic examination included assessing the patient’s general health, nasal function, and any underlying conditions that might contribute to or complicate CRS. Additionally, radiological imaging (CT) and endoscopic evaluation were performed by the same examiner to objectively confirm the diagnosis and assess the extent of sinus involvement.

CT imaging of the sinuses was performed on all patients, as per standardized protocols. Radiological findings included the presence of inflammation of the paranasal sinuses. The Lund-Mackay scoring system was used to quantify the degree of sinus involvement (mucosal thickening, sinus opacification).

Nasal endoscopy was performed and included the findings like purulent mucus or edema in the middle meatus or ethmoid region, presence of polyps in the nasal cavity or middle meatus, evaluation of sinus ostia patency, and mucosal changes. The Lund-Kennedy scoring system was used to assess the severity of mucosal inflammation, polyps, and obstruction.

## Results

Gender distribution

**Figure 1:**
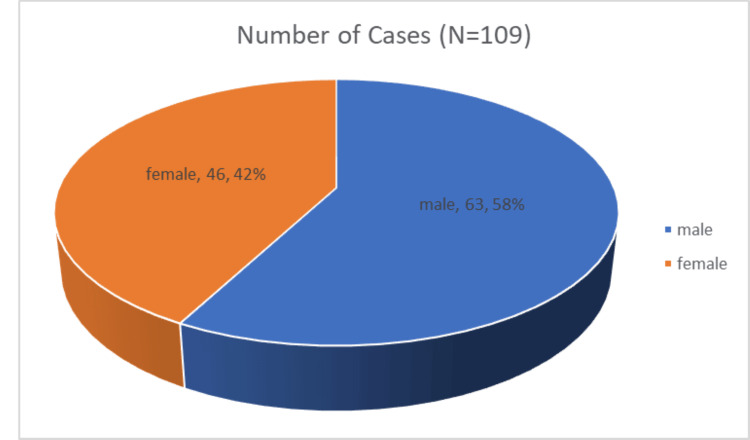
Gender distribution (N=109)

Out of the 109 cases included in the study, 63 (57.80%) were male patients, while 46 (42.20%) were female patients, reflecting a slight male predominance in the study population. This distribution is illustrated in Table 1 and Figure 1.

**Table 1:** Gender distribution

Gender	No. of cases (n=109)	Percentage
Male	63	57.80%
Female	46	42.20%

Age distribution

**Figure 2:**
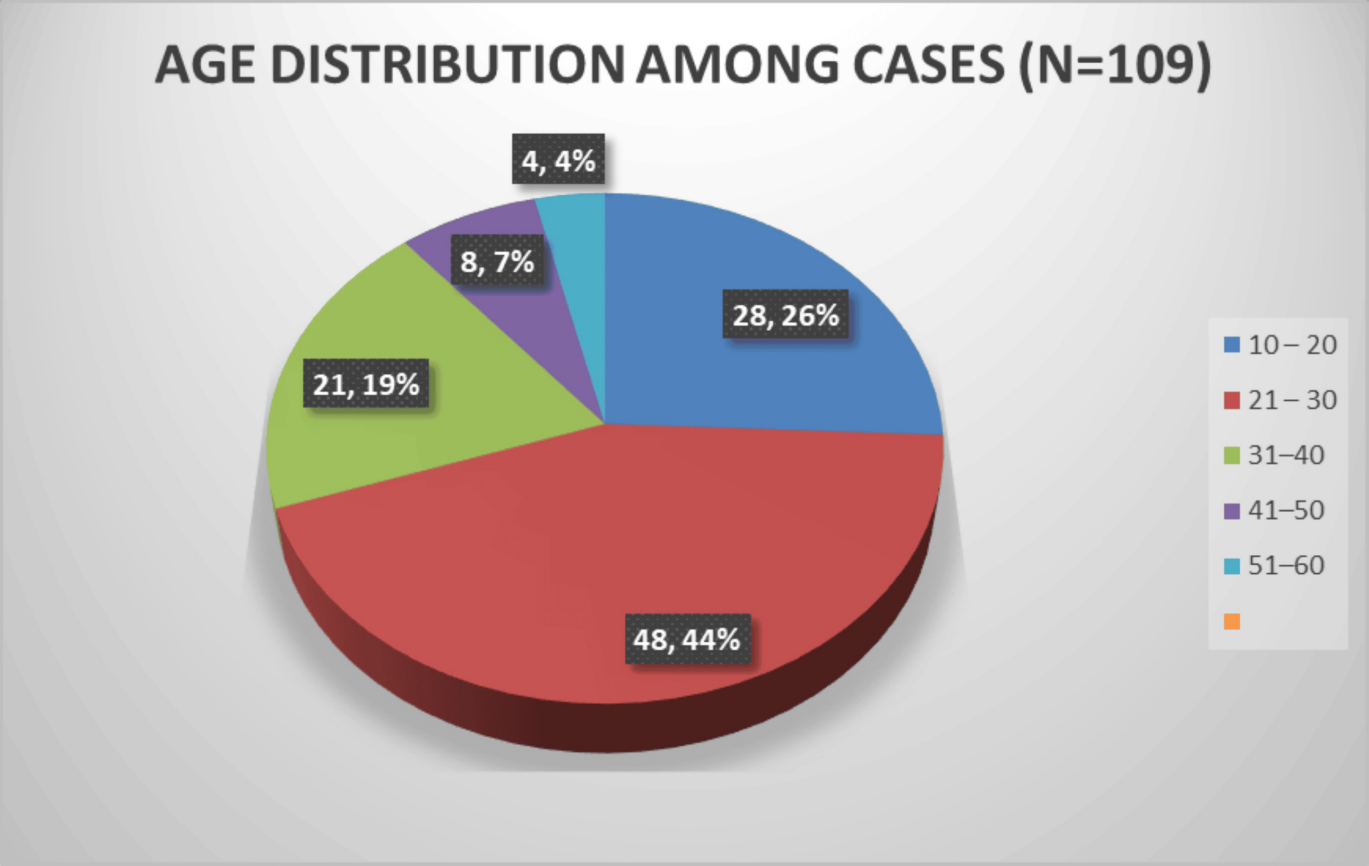
Age distribution among cases (N=109)

The age distribution of the study population ranged from 10 to 60 years. The highest prevalence was observed in the 21-30 years age group, accounting for 48 cases (44.03%). This was followed by the 10-20 years group (pediatric age group) with 28 cases (25.69%). The distribution is detailed in Table 2 and Figure 2.

**Table 2:** Age distribution

Age in years	Number	​ Percentage
10 – 20 (pediatric age group)	28	25.69%
21 – 30	48	44.03%
31–40	21	19.26%
41–50	8	07.34%
51–60	4	03.67%
Total	109	100%

**Figure 3:**
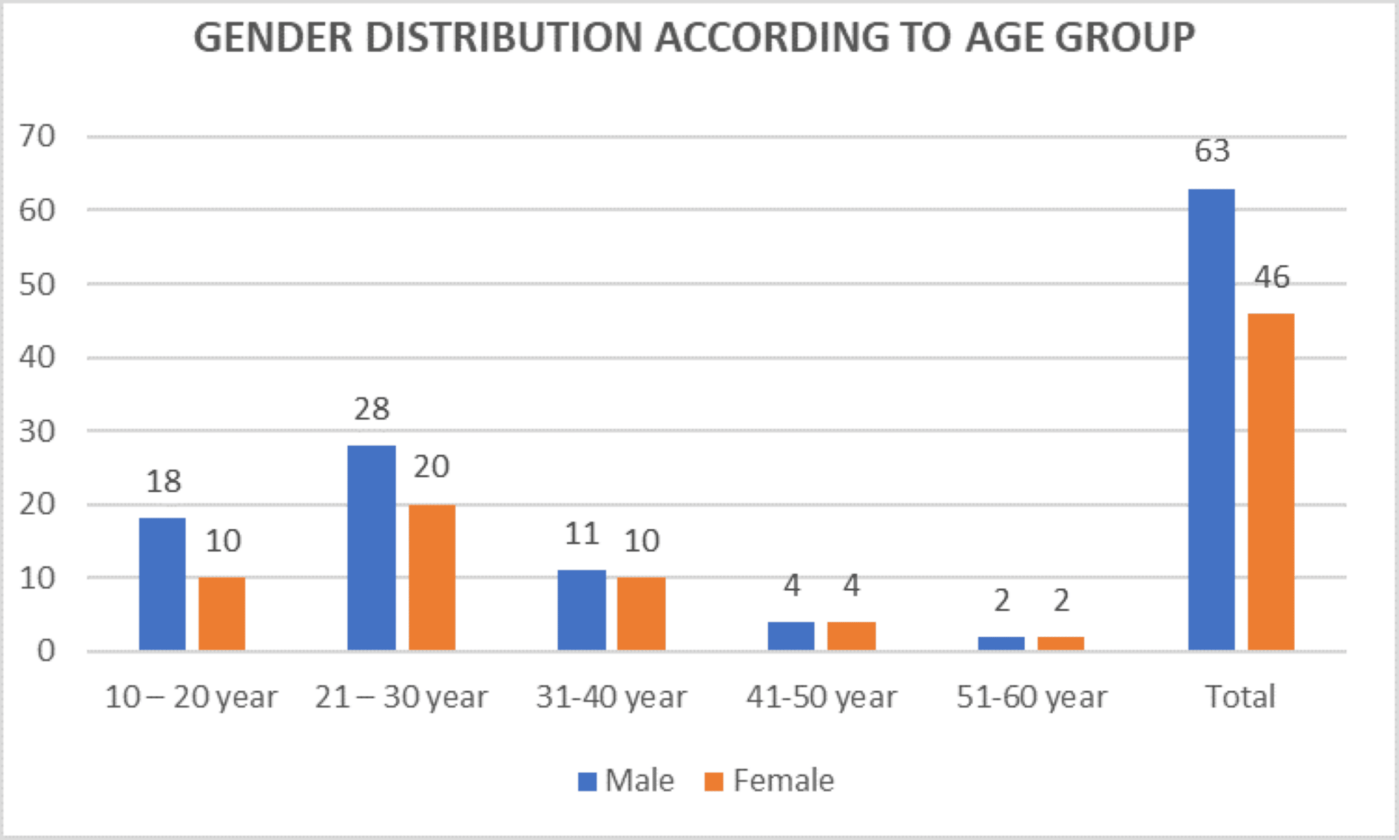
Gender distribution according to age group

The distribution of cases by age and gender shows that the highest number of cases in both male and female patients falls within the 21-30 years age group. The data are presented in Table 3 and Figure 3.

**Table 3:** Gender distribution according to age group

Age group	Male	Female
10 – 20 year (pediatric age group)	18	10
21 – 30 years	28	20
31-40 years	11	10
41-50 years	4	4
51-60 years	2	2
Total	63	46

**Figure 4:**
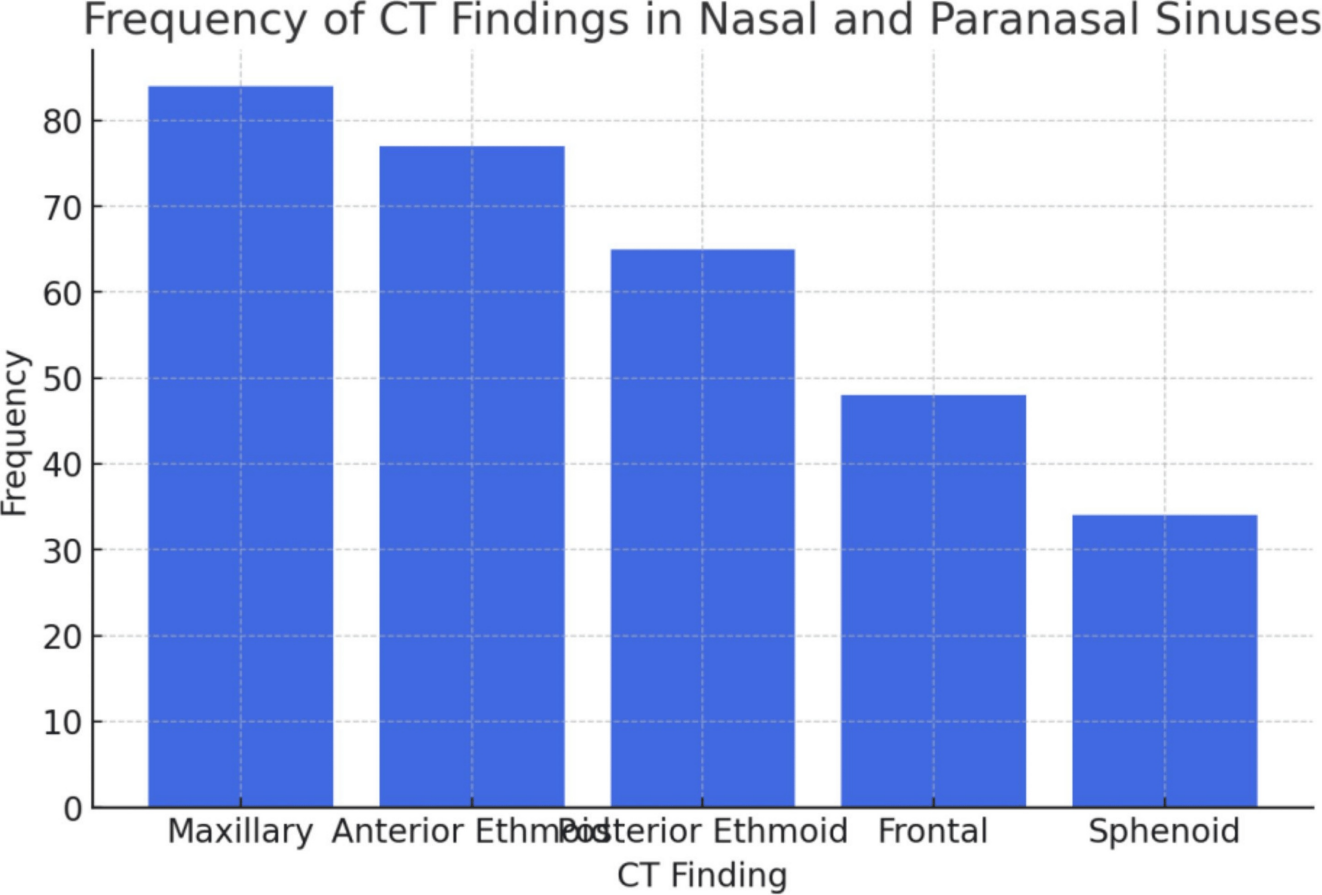
CT findings in study subjects

CT imaging findings revealed a distinct pattern of sinus involvement, as shown in Table 4 and Figure 4. The maxillary sinus was the most commonly affected, observed in 84 cases (77.1%), followed by the anterior ethmoid in 77 cases (70.6%).

**Table 4:** Radiological findings in study subjects

CT finding	Frequency of finding	Percentage
Maxillary	84	77.1%
Anterior ethmoid	77	70.6%
Posterior ethmoid	65	59.6%
Frontal	48	44.0%
Sphenoid	34	31.2%

**Figure 5:**
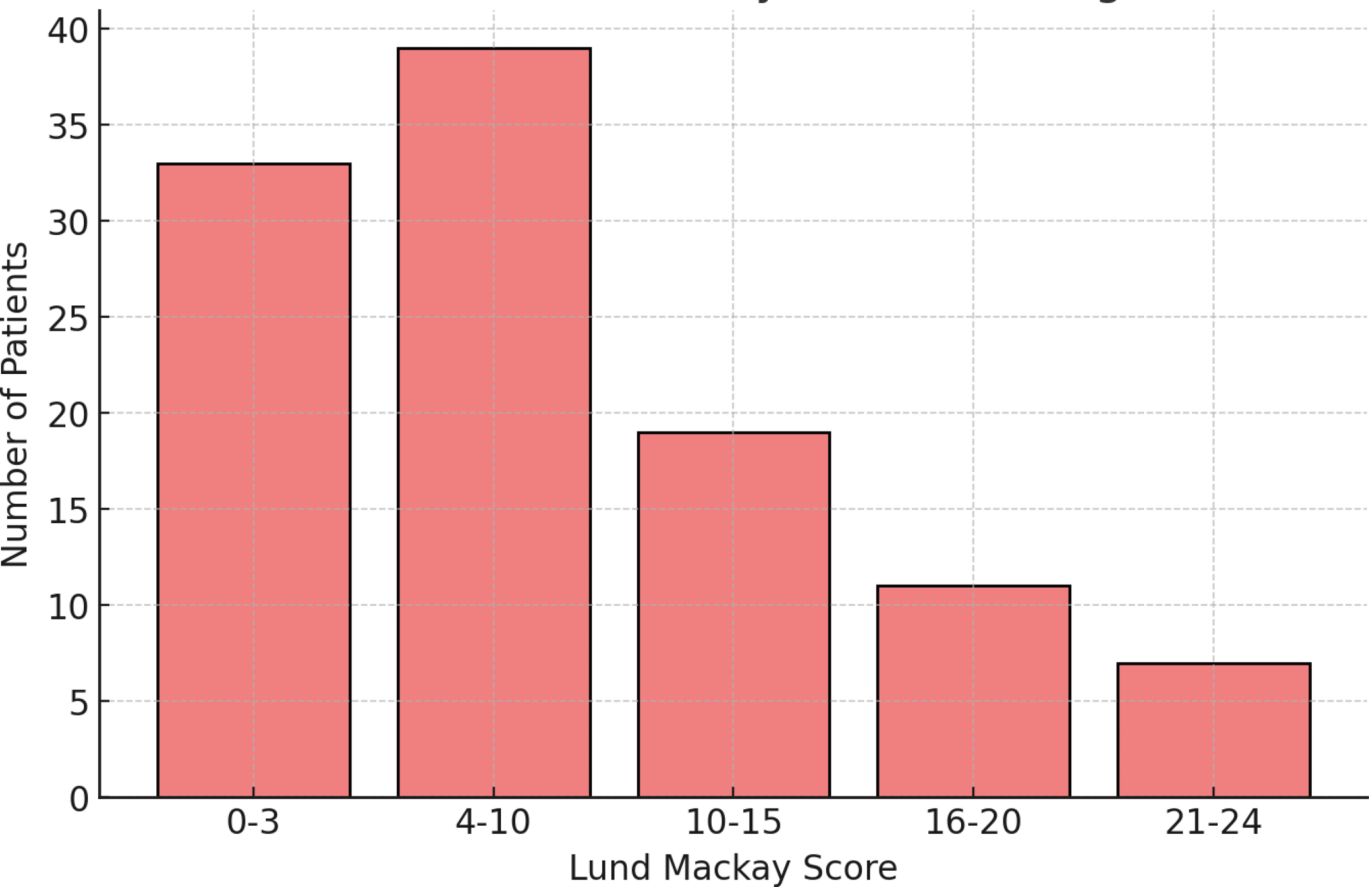
Distribution of Lund-Mackay score among patients

The distribution of Lund-Mackay scores among the patients is provided in Table 5 and Figure 5. Scores of 4 or more were considered positive, with 76 patients (69.7%) testing positive.

**Table 5:** Distribution of Lund-Mackay score among patients

Score	Patients
0-3	33
4-10	39
11-15	19
16-20	11
21-24	7
Total	109

**Figure 6:**
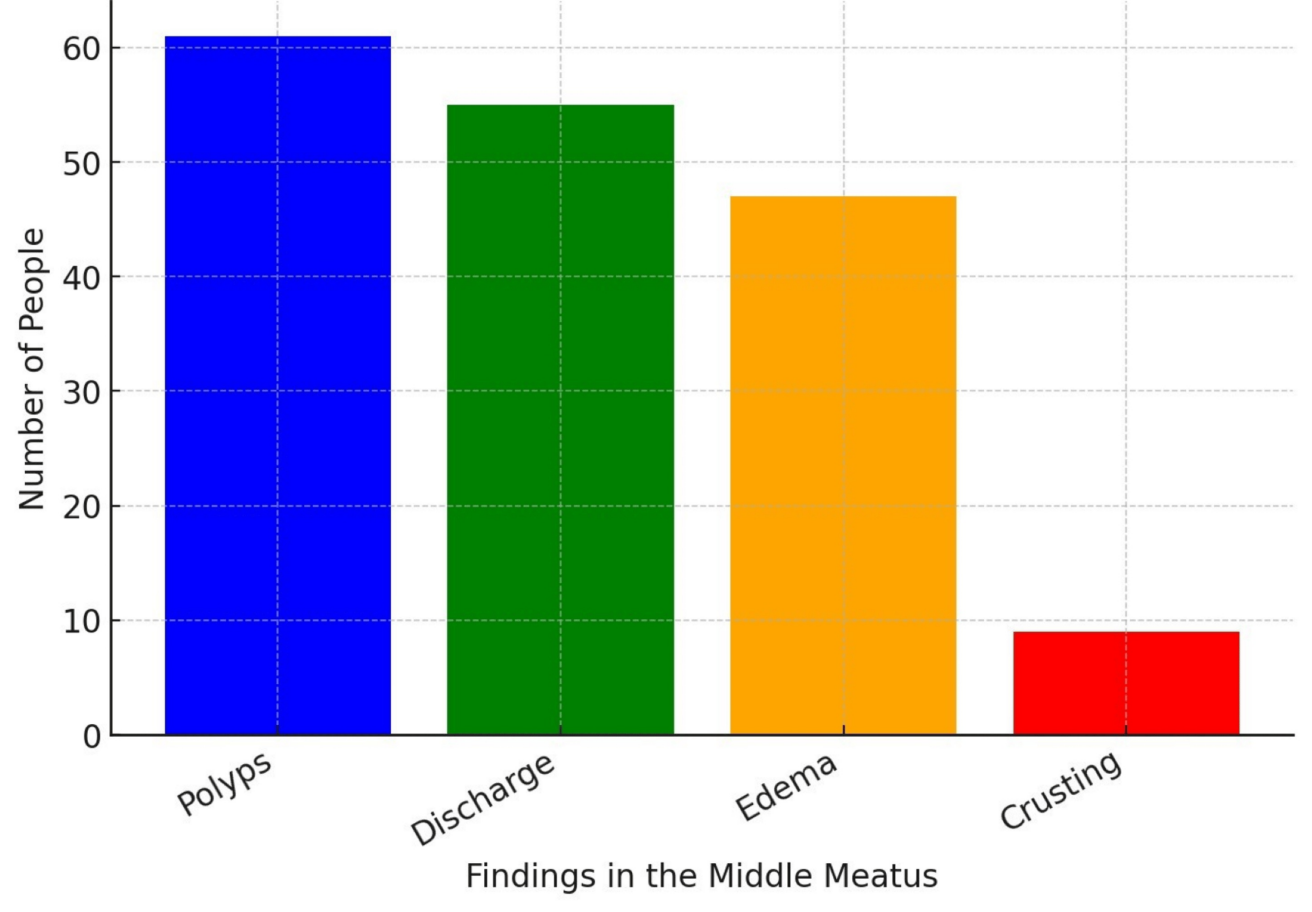
Findings in middle meatus

Findings from nasal endoscopy are summarized in Table 6 and Figure 6. Polyps were the most common observation, present in 61 cases (56.0%), followed by discharge (55 cases, 50.5%).

**Table 6:** Endoscopic DNE findings in study subjects DNE: diagnostic nasal endoscopy

Findings	No. of people
Polyps in the middle meatus	61
Discharge in the middle meatus	55
Edema of the middle meatus	47
Crusting in the middle meatus	9

**Figure 7:**
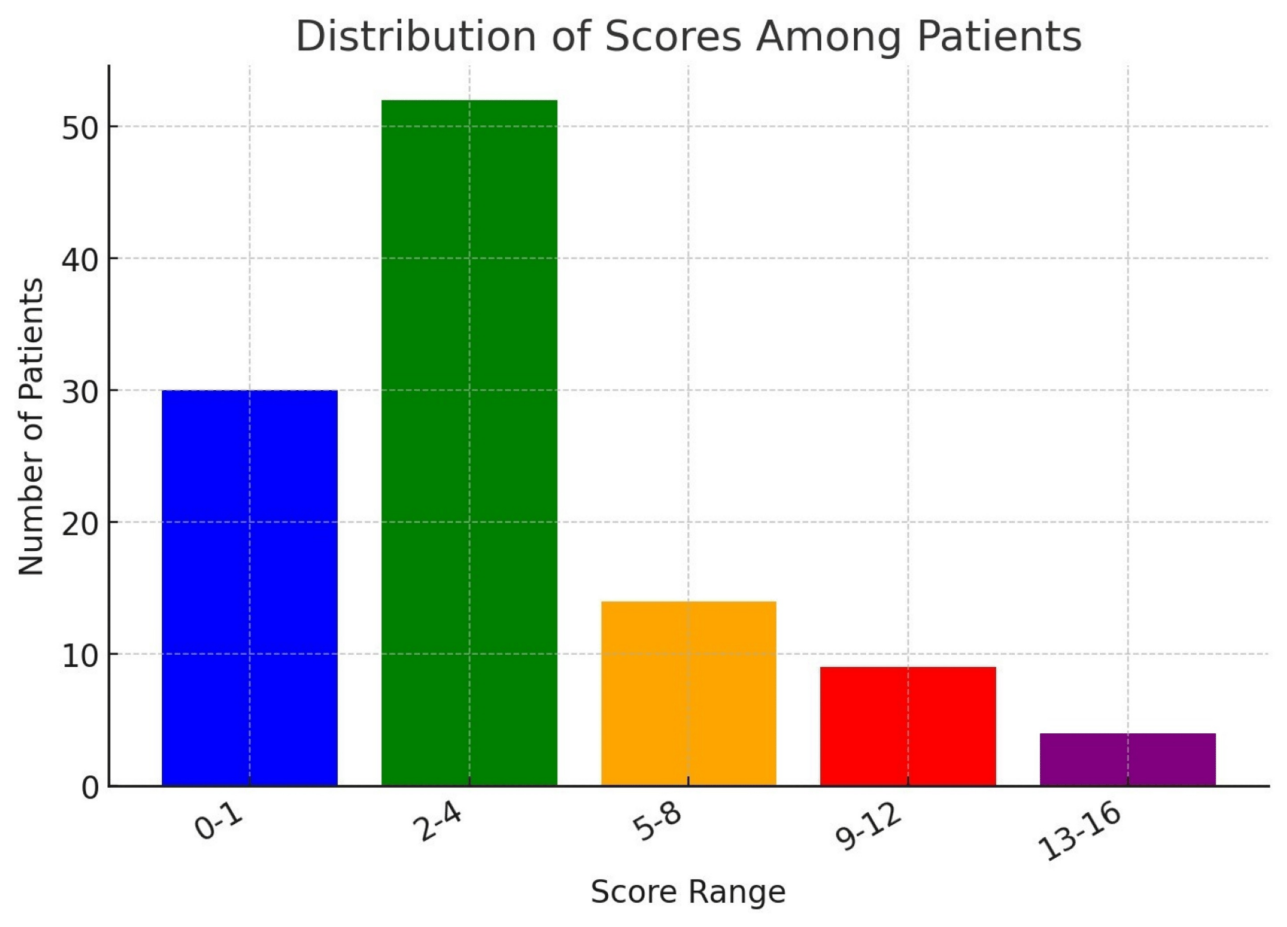
Distribution of Lund-Kennedy scores among patients

Out of 109 patients, 79 (72.5%) tested positive for chronic rhinosinusitis with Lund-Kennedy scores above 1, while 30 patients (27.5%) tested negative with scores ranging from 0 to 1 (Table 7, Figure 7). The positive cases had scores of 2 or more, indicating the presence of the condition with varying degrees of severity.

**Table 7:** Distribution of Lund-Kennedy scores among patients

Score	No. of patients
0-1	30
2-4	52
5-8	14
9-12	9
13-16	4

Table 8 illustrates the comparison between radiological and endoscopic findings, providing insight into their diagnostic accuracy. The true positive cases (71) demonstrate strong agreement between both modalities in detecting the condition, while true negative cases (25) confirm the absence of the disease. However, some discrepancies exist, with five false-positive cases and eight false-negative cases, indicating occasional mismatches between the two diagnostic methods.

**Table 8:** Comparison of radiological and endoscopic findings TP: true positive; FN: false negative; FP: false positive; TN: true negative

Category	Radiological positive	Radiological negative	Total
Endoscopic positive	71 (TP)	8 (FN)	79
Endoscopic negative	5 (FP)	25 (TN)	30
Total	76	33	109

Table 9 shows the diagnostic performance metrics highlighting the effectiveness of the test in identifying positive and negative cases. With a sensitivity of 93.42%, the test demonstrates a strong ability to detect true positive cases, ensuring that most affected individuals are correctly identified. The specificity of 75.75% indicates moderate accuracy in identifying true negatives, meaning some false positives may still occur. The PPV of 89.33% suggests a high likelihood that a positive test result truly indicates the condition, while the NPV of 83.33% reflects a good probability that negative results are accurate. Finally, the Youden Index of 69.17% represents the overall effectiveness of the test, balancing both sensitivity and specificity to ensure reliable diagnostic performance.

**Table 9:** Comparison oF DNE considering CT as a gold standard for objective diagnosis of CRS DNE: diagnostic nasal endoscopy; CRS: chronic rhinosinusitis

Measure	Percentage
Sensitivity	93.42%
Specificity	75.75%
Positive predictive value	89.33%
Negative predictive value	83.33%
Yoden Index number	69.17%

The statistical analysis revealed a significant correlation between endoscopic and radiological findings (χ² = 55.20, p < 0.00000001), highlighting the reliability of these diagnostic approaches. DNE demonstrated a sensitivity of 93.42% and a specificity of 75.75%. The PPV for detecting significant sinus disease was 89.33%, while the NPV was 83.33%. These results confirm that while DNE is highly effective for identifying mucosal abnormalities, it may lack the detailed anatomical resolution provided by CT, underscoring the complementary nature of these diagnostic tools.

## Discussion

The findings of this study reinforce the diagnostic value of DNE in the assessment of CRS, aligning with existing literature that highlights its high sensitivity, including among pediatric patients. DNE enables direct visualization of the nasal mucosa, detection of polyps, mucopurulent secretions, and structural abnormalities within the accessible nasal cavity. Wuister et al. and Bhattacharyya et al. previously emphasized the critical role of DNE in the initial clinical evaluation of CRS, particularly in cases with nasal polyposis, where mucosal changes are readily observable [12,13].

Furthermore, Mahajan et al. identified key anatomical landmarks such as the hiatus semilunaris, infundibulum, frontal recess, and sphenoethmoidal recess, which are areas through which the majority of sinuses drain [14]. Visualization of these regions during endoscopy allows clinicians to detect signs of localized inflammation or obstruction, thereby inferring pathology in adjacent sinuses. This supports the utility of DNE as a front-line diagnostic tool in CRS.

However, despite its strengths, DNE is inherently limited in its ability to evaluate deeper sinus cavities, such as the posterior ethmoid, sphenoid, and frontal sinuses, especially in cases without obvious intranasal findings. In such instances, computed tomography (CT) remains the gold standard for comprehensive assessment. Bolger et al. and Parmar et al. underscored the complementary roles of DNE and CT, where DNE allows for dynamic, real-time evaluation of the nasal cavity and mucosa, while CT provides detailed anatomical mapping, identification of anatomical variants, and staging of sinus disease [15,16]. CT is especially valuable in surgical planning and in cases where DNE findings are inconclusive or overlap with other conditions such as allergic rhinitis or neoplasms, as noted by Poto et al. [17].

Moreover, DNE may be subject to interobserver variability, and its diagnostic accuracy can depend significantly on operator experience and equipment quality. In resource-limited settings, DNE offers a cost-effective alternative when CT is unavailable, as supported by Acharia et al., though this must be balanced against the risk of underdiagnosing disease in less accessible sinus regions [18].

Limitations

This study has several limitations. First, it was conducted at a single center, which may limit the generalizability of the findings. The sample size, although adequate for preliminary analysis, may not fully capture the variability in CRS presentation across broader populations. Additionally, the study did not include a blinded comparison between DNE and CT scan findings, which could introduce bias. Another limitation is the lack of long-term follow-up data to assess outcomes based on diagnostic modality, and no histopathological confirmation was used in ambiguous cases.

## Conclusions

DNE is a valuable, minimally invasive tool for diagnosing CRS, offering high sensitivity in detecting mucosal inflammation. However, CT remains essential for a complete anatomical assessment, particularly in complex or refractory cases. A combined diagnostic approach utilizing both modalities is recommended for optimal CRS management.
